# Gamma-Glutamyl Transferase Levels in Patients with Acute Ischemic Stroke

**DOI:** 10.1155/2014/170626

**Published:** 2014-08-18

**Authors:** Nurbanu Gurbuzer, Eren Gozke, Zeliha Ayhan Basturk

**Affiliations:** Department of Neurology, FSM Teaching and Research Hospital, E-5 Uzeri, Bostanci, 34744 Istanbul, Turkey

## Abstract

*Objective*. The aim of this study was to investigate the relationship between gamma-glutamyl transferase (GGT) levels, cerebrovascular risk factors, and distribution of cerebral infarct areas in patients with acute ischemic stroke (AIS). *Patients and Methods*. Sixty patients with AIS and 44 controls who had not cerebrovascular disease were included in the study. The patients were divided into four groups according to the location of the infarct area and evaluated as for GGT levels and the presence of diabetes mellitus (DM), hypertension (HT), and hyperlipidemia (HL). *Results*. The frequency of DM, HT, and HL and gender distributions were similar. The mean GGT levels were significantly higher in patients with AIS and those with relatively larger areas of infarction (*P* < 0.05). Increased mean GGT levels were found in the subgroup with hypertension, higher LDL-cholesterol, and triglyceride levels among cases with AIS (*P* < 0.05). *Conclusion*. Higher GGT levels in AIS patients reinforce the relationship of GGT with inflammation and oxidative stress. The observation of higher GGT levels in patients with relatively larger areas of infarction is indicative of a positive correlation between increases in infarct areas and elevated GGT levels.

## 1. Introduction

Gamma-glutamyl transferase (GGT) mediates intracellular intake of extracellular glutathione which is an important component of antioxidant mechanisms. Glutathione is produced during normal metabolic processes and plays an important role in the protection of cells against oxidative stress. GGT has been used for years as an index of hepatic dysfunction and marker of alcohol use [1, 2]. In population-based studies, after exclusion of alcohol consumption, a positive correlation has been demonstrated between higher GGT levels and advanced age, male gender, increases in body mass index (BMI), smoking, sedentary life style, hypertension, tachycardia, hyperglycemia, increased LDL-cholesterol, and decreased HDL-cholesterol levels, hypertriglyceridemia, menopause, and oral contraceptive use [3, 4].

In this study, our aim was to investigate the relationship between serum GGT levels and several risk factors for cerebrovascular disease (CVD) and also distribution of cerebral infarct areas in patients with acute ischemic stroke (AIS).

## 2. Material and Method

Sixty patients hospitalized with the diagnosis of AIS and 44 CVD-naïve individuals who consulted to neurology polyclinic for other reasons were investigated. Patients with a history of chronic liver or renal disease, endocrine, and autoimmune diseases other than diabetes, alcoholics, smokers, those who had undergone surgical interventions related to coronary, carotid, or extremity arteries, or users of drugs which might alter GGT test results (lipid-lowering drugs, antibiotics) were excluded from the study. However diabetic and/or hypertensive patients continued to use the drugs for regulation of their glycemic state and blood pressure levels. The patients with newly onset angina, myocardial infarction, and advanced heart failure were not enrolled in the study. Levels of liver enzymes, bilirubin, and hepatic markers of all cases were within normal limits. Blood samples of AIS patients were obtained within twenty-four hours after stroke.

According to Bamford classification, stroke patients were divided into 4 groups based on the infarct area as total anterior circulation infarcts (TACI), partial anterior circulation infarcts (PACI), lacunar infarcts (LACI), and posterior circulation infarcts (POCI). In the patient and the control groups, study participants were evaluated as for the presence of diabetes mellitus (DM), hypertension (HT), hyperlipidemia (HL), and alterations in serum GGT levels. GGT was analyzed using a spectrophotometric method, and values ranging between 7 and 60 U/L were considered to be within physiologic limits.

In the statistical analysis Student's *t*-test, independent samples *t*-test, Mann Whitney *U* test, chi-square test, and Kruskal-Wallis test were performed. The significance level was evaluated at *P* < 0.05.

## 3. Results

Demographic data are seen in Table 1. A significant difference was not found between groups regarding mean ages and gender distribution. The frequencies of hypertension, diabetes mellitus, and hyperlipidemia in the cases with AIS and controls were comparable (88.3 versus 88.6%, 38.3 versus 38.6%, and 36.6 versus 40.9%, resp.). Mean GGT level in the AIS group was found to be significantly higher relative to the control group (23.3 ± 11.8 versus 15.0 ± 5.7 IU/L; *P* < 0.000). The cut-off value for GGT was calculated as 26.4 IU/L. Mean GGT level in the AIS group did not differ between female and male cases (23.1 ± 13.9 versus 23.5 ± 9.9 IU/L, resp.; *P* > 0.05). The lowest and the highest GGT levels were detected in the LACI and PACI groups, respectively. Mean GGT levels were significantly higher in TACI, PACI, and POCI groups than in LACI group (Table 2). Increased GGT level was found in the subgroup with hypertension, higher LDL-cholesterol, and triglyceride levels among cases with AIS (Table 3).

## 4. Discussion

Although gamma-glutamyl transferase is mostly found within cytosoles, it is also present on cellular membrane in considerable amounts and plays a role in intracellular ingress of amino acids and peptides in the form of *γ*-glutamyl peptides. Glutathione is its most important substrate. This tripeptide is a thiol derivative which is the most important nonprotein intracellular component and functions as a primary determinant of cellular redox mechanisms. In conditions giving rise to cellular stress, intracellular glutathione levels decrease. Decreased intracellular glutathione levels induce formation of GGT enzyme so as to maintain preexisting levels. Increased oxidative stress enhances requirement for glutathione. In the presence of inadequate amounts of glutathione, oxidative stress exerts more harmful effects [5–7]. The mechanism of the relationship between cardiovascular and cerebrovascular risk factors and GGT level is not fully known. According to a currently entertained theory, oxidative stress and related decrease in glutathione levels induce activity of GGT. Independent of alcohol consumption and presence of a liver disease, the predictive role of GGT activity in the development of new cases of diabetes, hypertension, and ischemic stroke has been established [8–12].

The Vorarlberg Health Monitoring and Promotion Program (VHM&PP) study conducted by Ruttmann et al. in Austria is the largest scale prospective study performed up to date [13]. This epidemiologic study has investigated the association between GGT and cardiovascular mortality. In this survey study an independent but significant association between GGT levels and cardiovascular mortality in both female and male cohorts was found. In the male patient cohort, a significant correlation between GGT and cardiovascular disease and ischemic and hemorrhagic stroke was present. In the same study, the correlation between GGT and cardiovascular disease was observed, but a statistically significant correlation between GGT levels and stroke (both hemorrhagic and ischemic types) could not be detected. Besides, prognostic significance of GGT was more prominently observed in patients younger than 60 years of age. Also in our study GGT levels were statistically significantly higher in the ischemic stroke group relative to the control group. However distribution of increased GGT values in the female and male patient groups did not differ significantly.

A multicenter prospective epidemiologic study (CARDIA, The Coronary Artery Risk Development in Young Adults) investigated 5115 individuals aged between 17 and 35 years. CARDIA study revealed correlations among normal GGT levels, diabetes, and hypertension [14]. The investigators also reported potential role of oxidative stress as a risk factor for the development of diabetes and hypertension and concluded that GGT is a sensitive early stage predictor of oxidative stress. In our study, a statistically significant difference was not observed between patients with or without diabetes; however, significant increases in GGT levels were noted in hypertensive patients. Besides significantly higher GGT levels were detected in patients with increased LDL-cholesterol and triglyceride levels.

D'Ambrosio et al. reported that increased gamma-glutamyl transferase levels predict functional impairment in elderly adults after ischemic stroke [15]. Korantzopoulos et al. also found positive correlation between serum gamma-glutamyl transferase and acute ischemic nonembolic stroke in the elderly subjects [16]. The role played by oxidative stress, subclinical inflammation in the pathophysiology of cardiovascular diseases, and development of stroke is already acknowledged. Mechanisms related to oxidative stress and subclinical inflammation can account for the role of GGT in the development of cerebrovascular disease [5, 17–20]. Increased GGT levels can play a pathogenetic role in the evolution and instability of atherosclerotic plaques in different vascular regions [21].

In our study, significantly higher GGT levels were detected in the AIS group relative to the control group. Besides, statistically significantly higher levels of GGT in cases with hypertension, increased LDL-cholesterol, and triglyceride levels suggest the role of oxidative stress. GGT might increase secondary to arterial wall inflammation and resultant arterial wall thickening, and higher GGT levels might protect arterial wall against oxidative stress as well.

In conclusion, higher GGT levels in AIS patients relative to the control group reinforce the relationship of GGT with inflammation and oxidative stress. Detection of relatively higher levels of GGT in AIS patients with hypertension, increased LDL-cholesterol, and triglyceride levels indicates the presence of a positive correlation between GGT levels, oxidative stress, and inflammation. When compared with the LACI group of patients with relatively smaller lacunar infarcts, observation of higher GGT levels in TACI, PACI, and POCI groups with relatively larger areas is indicative of a positive correlation between increases in infarct areas and elevated GGT levels. Future studies can better reveal the possible role of GGT in the prediction of oxidative stress and mild degrees of chronic inflammation.

## Figures and Tables

**Table 1:** Demographic data of the cases with AIS and the control group.

	AIS *n* = 60	Control *n* = 44	*P*
Age (mean ± SD)	71.7 ± 9.9	69.5 ± 8.7	0.065
Age range	51–90	50–82	

	AIS *n* (%)	Control *n* (%)	*P*

Gender			
Male	32 (53%)	19 (43%)	0.273
Female	28 (47%)	25 (57%)	

Student's *t*-test.

AIS: acute ischemic stroke.

**Table 2:** Mean GGT levels in cases with AIS according to infarction area.

Infarction area	*n* (%)	GGT mean ± SD (IU/L)	*P*
LACI	15 (25.0%)	16.0 ± 6.1	0.044*
PACI	21 (35.0%)	26.7 ± 15.3	
POCI	16 (26.7%)	24.4 ± 11.1	
TACI	8 (13.3%)	25.8 ± 3.1	

Kruskal-Wallis test.

LACI: lacunar infarctions, PACI: partial anterior circulation infarctions, POCI: posterior circulation infarctions, and TACI: total anterior circulation infarctions.

∗Statistically significant (mean GGT level in the LACI group was significantly lower than other groups).

**Table 3:** Mean GGT levels in cases with AIS according to gender and risk factors.

	*n*	GGT mean ± SD (IU/L)	*P*
Gender			
Female	28	23.18 ± 13.98	0.918
Male	32	23.50 ± 9.910	
LDL cholesterol			
<129	24	17.25 ± 8.330	0.00*
>129	36	27.42 ± 12.21	
Triglyceride			
>150	27	27.00 ± 8.350	0.03*
<150	33	20.36 ± 13.50	
HDL cholesterol			
>40	15	27.13 ± 17.87	0.155
<40	45	22.09 ± 8.970	
Total cholesterol			
>200	22	23.86 ± 7.580	0.801
<200	38	23.05 ± 13.83	
Diabetes mellitus			
(−)	37	21.49 ± 8.620	0.124
(+)	23	26.35 ± 15.50	
Hypertension			
(−)	7	14.14 ± 2.670	0.028*
(+)	53	24.57 ± 12.08	

Chi-square test.

∗Statistically significant.
